# Accelerating the Smith-Waterman algorithm with interpair pruning and band optimization for the all-pairs comparison of base sequences

**DOI:** 10.1186/s12859-015-0744-4

**Published:** 2015-10-06

**Authors:** Daiki Okada, Fumihiko Ino, Kenichi Hagihara

**Affiliations:** Graduate School of Information Science and Technology, Osaka University, 1-5 Yamadaoka, Suita, 565-0871 Japan

**Keywords:** Local alignment, Smith-Waterman algorithm, All-pairs comparison, Pruning

## Abstract

**Background:**

The Smith-Waterman algorithm is known to be a more sensitive approach than heuristic algorithms for local sequence alignment algorithms. Despite its sensitivity, a greater time complexity associated with the Smith-Waterman algorithm prevents its application to the all-pairs comparisons of base sequences, which aids in the construction of accurate phylogenetic trees. The aim of this study is to achieve greater acceleration using the Smith-Waterman algorithm (by realizing interpair block pruning and band optimization) compared with that achieved using a previous method that performs intrapair block pruning on graphics processing units (GPUs).

**Results:**

We present an interpair optimization method for the Smith-Waterman algorithm with the aim of accelerating the all-pairs comparison of base sequences. Given the results of the pairs of sequences, our method realizes efficient block pruning by computing a lower bound for other pairs that have not yet been processed. This lower bound is further used for band optimization. We integrated our interpair optimization method into SW#, a previous GPU-based implementation that employs variants of a banded Smith-Waterman algorithm and a banded Myers-Miller algorithm. Evaluation using the six genomes of Bacillus anthracis shows that our method pruned 88 % of the matrix cells on a single GPU and 73 % of the matrix cells on two GPUs. For the genomes of the human chromosome 21, the alignment performance reached 202 giga-cell updates per second (GCUPS) on two Tesla K40 GPUs.

**Conclusions:**

Efficient interpair pruning and band optimization makes it possible to complete the all-pairs comparisons of the sequences of the same species 1.2 times faster than the intrapair pruning method. This acceleration was achieved at the first phase of SW#, where our method significantly improved the initial lower bound. However, our interpair optimization was not effective for the comparison of the sequences of different species such as comparing human, chimpanzee, and gorilla. Consequently, our method is useful in accelerating the applications that require optimal local alignments scores for the same species. The source code is available for download from http://www-hagi.ist.osaka-u.ac.jp/research/code/.

## Background

Pairwise sequence alignment identifies similar regions between two biological sequences (such as between nucleotide and protein sequences) and is useful for analyzing functional, structural, and evolutional relationships between the two. Such alignment algorithms can be classified into two groups: global and local alignment algorithms. The former produces an end-to-end alignment of sequences and the latter produces alignments that describe most similar regions within sequences. In particular, local alignment is useful for constructing a phylogenetic tree because it can identify regions in which mutations such as the insertions or deletions of nucleotides occurred in the evolutionary process.

The Smith-Waterman (SW) algorithm [1] is known as a dynamic programming scheme that yields the exact solutions for pairwise local alignments; its solutions produce similarity scores, similar regions in the sequences, and operations needed to match those similar regions. The SW algorithm consists of a matrix-filling phase and a backtracing phase. The matrix-filling phase computes the similarity scores of the arbitrary regions of sequences, and the backtracing phase identifies the local alignments that can be found from the highest-scoring matrix cell. Given the two sequences of lengths *m* and *n* (≥*m*), the time complexity of the SW algorithm is $\mathcal {O}(mn)$. Because the length of biological sequences can reach giga-base pairs (Gbp), many researchers have accelerated the SW algorithm using various hardware such as graphics processing units (GPUs) [2–5], single-instruction multiple-data (SIMD) enabled CPUs [6–8], field programmable gate arrays [9] and Xeon Phi [10]. Of these, GPUs [11] emerge as accelerators not only for graphics applications but also for general applications [12–14].

CUDAlign 1.0 [3] employed a GeForce GTX 280 GPU to parallelize the performance bottleneck part of the SW algorithm, namely, the matrix-filling phase. This tool first computed the local alignment score between the human chromosome 21 and the chimpanzee chromosome 22; it took 21 h to process the matrix-filling phase for sequences that were 47 Mbp long for the human chromosome and 33 Mbp long for the chimpanzee chromosome. To obtain complete alignment results, the tool was further extended to integrate the score-only Smith-Waterman algorithm with the Myers-Miller algorithm [15], which computes optimal global alignments in linear space. In addition, the tool realized efficient matrix filling with intrapair block pruning, after which it achieved a further acceleration of up to 51 % [16]. Meanwhile, SW# [5] implemented a parallel algorithm that can achieve further acceleration on two GPUs; that dual-GPU implementation aligned the human chromosome 21 with the chimpanzee chromosome 22 in 6.5 h on a GeForce GTX 690. Within 9 h, another multi-GPU implementation [17] aligned the human chromosome 1, which was 249 Mbp long, with the chimpanzee chromosome 1 that was 228 Mbp long by using 64 Tesla M2090 nodes.

To the best of our knowledge, the existing acceleration methods were designed for pairwise alignment. Consequently, we believe that further acceleration can be achieved for the all-pairs comparisons, which iterate pairwise alignments with all possible combinations of sequences to obtain accurate phylogenetic trees [18]. An all-pairs comparison requires ${N \choose 2}$ pairwise alignments, where *N* is the number of sequences to be investigated. Consequently, further acceleration of pairwise alignment is necessary.

We present an interpair optimization method for the SW algorithm that is useful for accelerating the all-pairs comparisons of sequences. According to the alignment results of several pairs, our method realizes efficient block pruning by computing a lower bound for another pair that has not yet been aligned. This lower bound is further used for band optimization [19], which restricts matrix filling within a certain anti-diagonal band. Consequently, our method is effective for investigating sequences that are highly similar to each other. This method is implemented on a dual-GPU system by extending the previous SW# implementation [5].

### Related work

Feng *et al.* [18] have presented a progressive method capable of constructing a phylogenetic tree from multiple sequences. Their method computes a distance matrix that represents the similarity between the sequences to be examined; such computation requires an all-pairs comparison of the sequences, but the comparison is processed sequentially without interpair pruning. In contrast, our method intends to accelerate the all-pairs comparison by interpair pruning.

Practical tools such as ClustalW [20] and T-Coffee [21] deploy a progressive method that solves the multiple-alignment problem using an approximated approach. These tools perform all-pairs comparisons with global alignments before processing the progressive method. ClustalW employs a fast approximate algorithm [22] to accelerate the all-pairs comparison. T-Coffee increases the accuracy of the solution by performing the all-pairs comparison not only with global alignment but also with local alignment, which is useful when compensating for multiple alignments but requires a long execution time [23]. In contrast to these tools, we realize interpair pruning for the all-pairs comparison by local alignment.

Sandes *et al.* [16] developed CUDAlign 2.1, which computes optimal local alignments in three phases, as Chao *et al.* [19] did: (1) the forward matrix-filling phase, which computes the highest alignment score and the ending alignment position, (2) the backward matrix-filling phase, which obtains the starting alignment position from the computed ending position, and (3) the reconstruction phase, which obtains the full alignment by applying the Myers-Miller algorithm [15] to subsequences between the starting and ending alignment positions. This tool also realized intrapair block pruning for efficient SW alignment. Their pruning method accelerates the matrix-filling phase by avoiding computation for matrix blocks that do not to improve a lower bound that has already been produced. That scheme successfully avoids 53.7 % of all matrix cell computations, which increases the alignment throughput from 28.6 GCUPS to 50.7 GCUPS on a GeForce GTX 560 for the human chromosome 21 and the chimpanzee chromosome 22. However, the lower bound is obtained from the ongoing pair to be aligned. Consequently, there is a limitation on the maximum number of matrix cells that can be pruned, for which the researchers provide a proof [16].

The same block pruning method was implemented by SW# [5], which parallelized the abovementioned three phases on a dual-GPU environment. Furthermore, SW# applies band optimization [19, 24] to the second and third phases, where the highest alignment score is known. Banded algorithms are useful to avoid computation for matrix cells being outside a certain anti-diagonal band. However, banded alignment algorithms assume that an optimal alignment exists within a pre-specified band. This assumption requires the highest alignment score to estimate the maximum number of insertions and deletions, which determines the width of the band. Without satisfying this assumption, optimal alignments cannot be obtained. With respect to the first phase, where the highest alignment score is unknown before computation, an alternative solution is to start matrix filling with an initial width and double the width until covering the full alignment. However, this iterative procedure is a time-consuming task for long sequences.

Many researchers have accelerated the SW algorithm using various computing platforms with parallelization and tuning techniques. Rognes *et al.* [8] accelerated the SW algorithm on multi-core CPUs. Their implementation achieved 106 GCUPS on two Xeon X5650 CPUs by achieving thread-based parallelization with multimedia SIMD instructions called Streaming SIMD Extensions (SSE) [25]. Liu *et al.* [10] implemented that SIMD-based algorithm on four Xeon Phi accelerators [26] and achieved 228 GCUPS at best. They also presented GPU-based implementations [27, 28] for homology searches that are useful for identifying, for example, amino acid sequences in the database that are most similar to any amino acid sequence given as a search query. Such homology searches were accelerated by exploiting the data parallelism inherent in the search process; different database sequences can be examined simultaneously. A similar GPU-based implementation was presented by Munekawa *et al.* [29], who extended the implementation by enabling idle GPU cycles to be explored, thus accelerating the homology search [13].

## Methods

Let $\mathcal {S} \triangleq \{ s_{1},s_{2},\cdots,s_{N} \}$ be a set of *N* sequences to be investigated. Let $\mathcal {P} \triangleq \left \{ \langle a,b \rangle ~\vert ~ s_{a},s_{b} \in \mathcal {S}, 1 \leq a<b \leq N \right \}$ be a set of ordered pairs. The goal of the all-pairs comparison of sequences is to compute the alignment scores and similar regions for all pairs in $\mathcal {P}$.

### Smith-Waterman algorithm

**Fig. 1:**
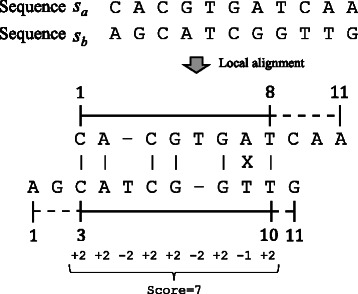
An example of local alignment. Two sequences *s*_*a*_ and *s*_*b*_ are aligned in this example. Alignment over sequence *s*_*a*_ is given by [*x*,*y*]=[ 1,8] while that over sequence *s*_*b*_ is given by [ *z*,*w*]=[ 3,10]. Notation “-” represents an empty symbol, which is skipped when computing *x*, *y*, *z* and *w*. In this example, the gap cost is *o*=−2 and the costs of a match and a mismatch are *α*=2 and *β*=−1, respectively

Let *s*_*a*_ and *s*_*b*_ be the sequences of lengths *m* and *n* (≥*m*) to be aligned. As shown in Fig. 1, the SW algorithm computes the alignment score of pair 〈*a*,*b*〉 according to the edit distance needed to convert *s*_*a*_ to *s*_*b*_. A pair of similar sequences produces a high alignment score.

Let *a*_*i*_ and *b*_*j*_ be the *i*-th symbol of sequence *s*_*a*_ and the *j*-th symbol of sequence *s*_*b*_, respectively. Let *S*(*i*,*j*) be the similarity function that represents the similarity between symbols *a*_*i*_ and *b*_*j*_: *S*(*i*,*j*)=*α* if *a*_*i*_=*b*_*j*_ and *β* otherwise. That is, *α* (≥0) represents the score when *a*_*i*_ matches *b*_*j*_, while *β* (<*α*) represents the unmatching score. As a scoring system, we assume affine gap penalty. The gap penalty of length *l* is given by *o*+*e*×(*l*−1), where *o* is the opening penalty and *e* is the extension penalty.

**Fig. 2:**
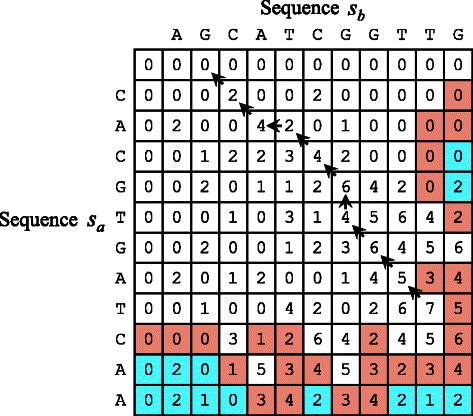
Smith-Waterman algorithm. The SW algorithm consists of matrix-filling and backtracing phases. Computation for the blue cells can be pruned during matrix filling. An orange cell is a triggering cell, which means pruning can spread to neighboring cells. Pruned matrix cells are typically located in either the lower half or the lower triangular matrix [16]

As shown in Fig. 2, the SW algorithm [1] consists of matrix-filling and backtracing phases. The former phase is based on dynamic programming that computes the maximum score of the alignment that ends at arbitrary positions. On the other hand, the latter phase identifies the most similar regions, namely, the subsequences that give the highest score according to the necessary replacement or insertion of symbols. The matrix-filling phase and backtracing phases require $\mathcal {O}(mn)$ time and $\mathcal {O}(m+n)$ time, respectively. Consequently, the former phase usually limits the entire performance.

**Fig. 3:**
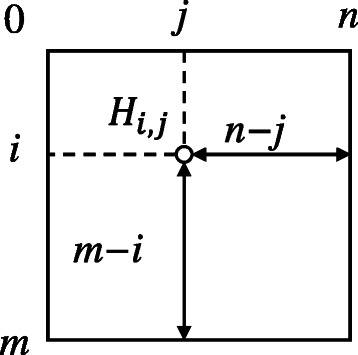
Geometrical representation of definitions. When computing matrix cell *H*_*i*,*j*_, there are *m*−*i* and *n*−*j* symbols left in sequences *s*_*a*_ and *s*_*b*_, respectively

Let *H*_*i*,*j*_ (0≤*i*≤*m*,0≤*j*≤*n*) be the maximum alignment score of subsequences ending with symbols *a*_*i*_ and *b*_*j*_. The score *H*_*i*,*j*_ is then given by the following equations [30]:

(1) $$\begin{array}{*{20}l} H_{i,j} &= \max \left\{ 0, E_{i,j}, F_{i,j}, H_{i-1,j-1} + S(i,j) \right\},  \end{array} $$

(2) $$\begin{array}{*{20}l} E_{i,j} &= \max \left\{ E_{i,j-1} - e, H_{i,j-1} - o \right\},  \end{array} $$

(3) $$\begin{array}{*{20}l} F_{i,j} &= \max \left\{ F_{i-1,j} - e, H_{i-1,j} - o \right\},  \end{array} $$

where *H*_*i*,0_=*E*_*i*,0_=*F*_*i*,0_=0 for all *i*, and *H*_0,*j*_=*E*_0,*j*_=*F*_0,*j*_=0 for all *j*. Figure 3 shows a geometrical representation of these definitions. Eqs. (1)–(3) indicate that a matrix cell (*i*,*j*) depends on its left, upper, and upper left neighbors, namely, (*i*−1,*j*), (*i*,*j*−1), and (*i*−1,*j*−1), respectively. Consequently, matrix cells are filled from the top left corner to the bottom right corner.

After filling all matrix cells, the backtracing phase is initiated at the highest-scoring cell and terminates on reaching a cell with a score of zero. This phase identifies the positions where a symbol must be replaced or an empty symbol must be inserted to obtain an alignment. A naive backtracing implementation cannot be used for long sequences, because it requires $\mathcal {O}(mn)$ space to perform backtracing. Therefore, the Myers-Miller algorithm [15], or a global alignment algorithm with linear space, is usually applied to subsequences between the starting and ending alignment positions. The Myers-Miller algorithm is based on the Hirschberg algorithm [31], which employs a recursive divide-and-conquer scheme to compute global alignments in $\mathcal {O}(m+n)$ space.

In the following discussion, let [*x*,*y*] be the most similar region in sequence *s*_*a*_, where *x* and *y* represent the starting and ending positions of the alignment, respectively, and 1≤*x*≤*y*≤*m*. For example, we have *x*=1 and *y*=8 for the case illustrated in Fig. 1. Note that a similar region is defined over the original sequence *s*_*a*_ so that neither the empty nor the replacing symbols are included in the region. Similarly to this definition over *s*_*a*_, which corresponds to the first entry of the ordered pair 〈*a*,*b*〉, let [*z*,*w*] be the similar region in sequence *s*_*b*_, where 1≤*z*≤*w*≤*n* (see Fig. 1).

### Interpair pruning

**Fig. 4:**
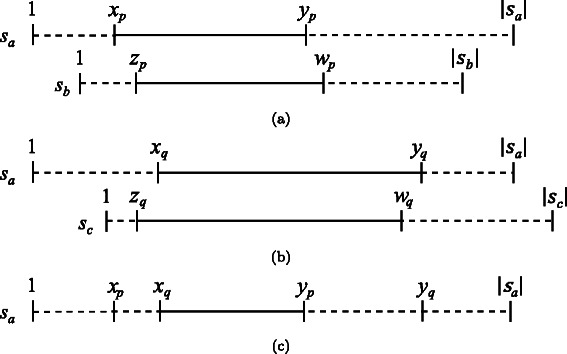
The common part of alignments. (**a**) Alignment for pair *p*=〈*a*,*b*〉, (**b**) alignment for pair *q*=〈*a*,*c*〉, and (**c**) their common part in sequence *s*_*a*_. This example assumes that *x*_*p*_<*x*_*q*_<*y*_*p*_<*y*_*q*_

Suppose that we have the alignment results for pair $p=\langle a,b\rangle \in \mathcal {P}$: the similar region is [*x*_*p*_,*y*_*p*_], *f*_*p*_ is the number of mismatched symbols, and *g*_*p*_ is the number of gaps. Similarly, the alignment results for pair $q=\langle a,c\rangle \in \mathcal {P}$, where *b*<*c*, are given as [*x*_*q*_,*y*_*q*_], *f*_*q*_, and *g*_*q*_. Using these results, our method accelerates the SW alignment for pair $r=\langle b,c \rangle \in \mathcal {P}$ by realizing efficient pruning during the matrix-filling phase. In the following discussion, we consider the common part of similar regions [*x*_*p*_,*y*_*p*_] and [*x*_*q*_,*y*_*q*_] (see Fig. 4).

#### Theorem 1.

Pairs *p*=〈*a*,*b*〉 and *q*=〈*a*,*c*〉, where *a*<*b*<*c*, do not have a common part in sequence *s*_*a*_ if and only if either *y*_*p*_<*x*_*q*_ or *y*_*q*_<*x*_*p*_ is satisfied. In other words, a common part exists if and only if *y*_*p*_≥*x*_*q*_ and *y*_*q*_≥*x*_*p*_ are satisfied. The common part in sequence *s*_*a*_ is then given by [max(*x*_*p*_,*x*_*q*_), min(*y*_*p*_,*y*_*q*_)].

Theorem 1 implies that our method cannot improve a lower bound for pair *r* if there is no common part between the aligned pairs *p* and *q* (i.e., if either *y*_*p*_<*x*_*q*_ or *y*_*q*_<*x*_*p*_). Consequently, we assume that *y*_*p*_≥*x*_*q*_ and *y*_*q*_≥*x*_*p*_ in the following discussion.

### Lower bound for the all-pairs comparison

The key idea of our interpair pruning method is to use a lower bound *L*_1_ on the alignment score of the common part [max(*x*_*p*_,*x*_*q*_), min(*y*_*p*_,*y*_*q*_)] as a lower bound *L* on the alignment score for pair *r*. Notice that the common part [max(*x*_*p*_,*x*_*q*_), min(*y*_*p*_,*y*_*q*_)] in sequence *s*_*a*_ corresponds to the subsequences of *s*_*b*_ and *s*_*c*_. Consequently, a lower bound *L*_1_ for the common part in sequence *s*_*a*_ can be used as a lower bound *L*_2_ on the alignment score for those subsequences. Because each subsequence is a part of sequences *s*_*b*_ and *s*_*c*_, the alignment score for pair *r*=〈*b*,*c*〉 is at least *L*_2_, which means that *L*_2_ can be used as a lower bound *L* for pair *r*=〈*b*,*c*〉.

**Fig. 5:**
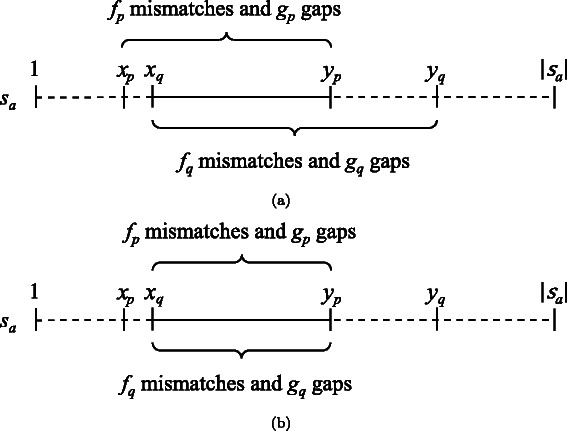
Matching and unmatching patterns for three sequences. Three operations can occur at every position on a sequence: a match, a replacement, and a gap insertion. Considering these operations, eight patterns can occur when considering sequences *s*_*a*_, *s*_*b*_, and *s*_*c*_. A lower bound on the alignment score for pair *r*=〈*a*,*c*〉 can be obtained by counting the number of matching symbols that commonly appear in each of the three sequences

**Fig. 6:**
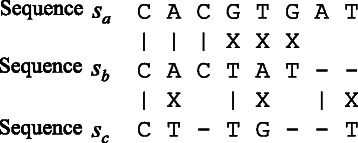
The worst case of the alignment score of the common part. (**a**) Alignment results on sequence *s*_*a*_ given by pairs *p*=〈*a*,*b*〉 and *q*=〈*a*,*c*〉. (**b**) The worst case that can be derived from the results. This example assumes that *x*_*p*_<*x*_*q*_<*y*_*p*_<*y*_*q*_

Figure 5 shows all matching and unmatching patterns that can be observed when aligning sequences *s*_*a*_, *s*_*b*_, and *s*_*c*_. As shown in that figure, a lower bound *L*_2_ for pair *r* can be computed by counting the number of matching symbols that commonly appear in all sequences *s*_*a*_, *s*_*b*_, and *s*_*c*_. Given the alignment results for pairs *p* and *q*, the number of matching symbols that commonly appear in all sequences *s*_*a*_, *s*_*b*_, and *s*_*c*_ is minimized if all *f*_*p*_+*f*_*q*_ mismatches and *g*_*p*_+*g*_*q*_ gaps of pairs *p* and *q* appear in the common part (see Fig. 6). Assuming this worst case, the total mismatch cost *F* and the total gap cost *G* are given by

(4) $$\begin{array}{*{20}l} F &= \beta \times \left(\,f_{p}+f_{q}\right),  \end{array} $$

(5) $$\begin{array}{*{20}l} G &= \max\left(o+e\times (g_{p}+g_{q}-1),o \times (g_{p}+g_{q})\right).  \end{array} $$

Consequently, a lower bound *L*_1_ on the alignment score for the common part can be given by

(6) $$ L_{1} = \max(0,~ \alpha \times M - F - G),   $$

where *M* is the number of matching symbols in the common part. This number *M* can be computed by subtracting the number of unmatching symbols from the length of the common part:

(7) $$\begin{array}{*{20}l} M &= \min\left(y_{p},y_{q}\right) - \max\left(x_{p},x_{q}\right) + 1 - \left(\,f_{p}+f_{q}\right).  \end{array} $$

Recall here that the common part does not include empty symbols because it is defined over the original sequence *s*_*a*_.

#### Corollary 1.

Eq. (6) is a lower bound on the score of the common part.

#### *Proof*.

Assume an alignment score *L*1′ of the common part such that *L*1′<*L*_1_. Because *L*_1_ assumes that all replacements and empty symbols exist in the common part (see Fig. 6), all of the remaining symbols in the common part are matching symbols. The total score of such remaining symbols must be a negative number to satisfy *L*1′<*L*_1_, which contradicts the assumption that *α*≥0. Thus, the conclusion is that *L*1′≥*L*_1_.

#### Theorem 2.

*L*_1_ is a lower bound on the alignment score for pair *r*. That is, *L* ^′^≥*L*_1_, where *L* ^′^ is the alignment score for pair *r*.

#### *Proof*.

Because local alignment produces the maximum alignment score starting from an arbitrary position and ending at an arbitrary position, a lower bound on the score of the common part is also a local bound on the alignment score for the entire region.

Our method starts the matrix-filling phase with *L*=*L*_1_ instead of *L*=0. The remaining procedure is exactly the same as that of the existing method [16].

Algorithm 1 describes how our method processes the all-pairs comparisons of sequences. Given *N* sequences, this algorithm outputs ${N \choose 2}$ pairwise alignment results for all the pairs of sequences. The ComputeLB() function at line 6 returns a lower bound according to Eq. (6). As shown in Algorithm 2, this function computes a lower bound for pair 〈*a*,*b*〉 from the alignment results of pairs 〈*c*,*a*〉 and 〈*c*,*b*〉, where *c*<*a*<*b*. The AlignPair() function at line 8 of Algorithm 1 returns a pairwise alignment result for pair 〈*a*,*b*〉 using the computed lower bound.





### Pairwise alignment with intrapair pruning

Our method extends an existing intrapair pruning method such that it can perform interpair pruning using the lower bound *L*=*L*_1_. This intrapair pruning method was originally presented by CUDAlign 2.1 [16], which can omit computation for any cell (*i*,*j*) that satisfies *i*≥⌈*m*/2⌉ or *i*≥*n*−*j* on a single GPU [16] (see Fig. 1). If *m*≤2*n*, the number of pruned cells is at most ⌊*m* *n*/2−*m* ^2^/8⌋; otherwise, it is at most ⌊*m* *n*−*n* ^2^/2⌋.

To describe this pruning method, we introduce the following definition:

#### Definition 1.

A triggering cell is defined as a matrix cell (*i*,*j*) such that it satisfies *H*_*i*,*j*_+*α*× max(*m*−*i*,*n*−*j*)<*L*, where *L* is a lower bound on the alignment score. As such a lower bound, the method uses the highest score that has already been produced before computing *H*_*i*,*j*_.

This definition indicates that none of the possible scores that can be obtained from a triggering cell is optimal although all symbols of uncompared subsequences *a*_*i*+1_ *a*_*i*+2_…*a*_*m*_ and *b*_*j*+1_ *b*_*j*+2_…*b*_*n*_ match. As shown in Fig. 3, the maximum number of such uncompared symbols is max(*m*−*i*,*n*−*j*). Therefore, the highest score that can be obtained from *H*_*i*,*j*_ is given by *H*_*i*,*j*_+*α*× max(*m*−*i*,*n*−*j*), which we mentioned in Definition 1.

According to Eq. (1), the following corollary can be obtained:

#### Corollary 2.

Computation for a matrix cell (*i*,*j*) can be pruned during matrix filling if all of (*i*−1,*j*), (*i*,*j*−1), and (*i*−1,*j*−1) are triggering cells or have already been pruned.

### Band optimization based on lower bound

The lower bound *L* can be used for not only interpair pruning but also band optimization [19]. That is, our band optimization method substitutes the lower bound *L* for the alignment score to estimate the minimum number of matches, which can be given by the ratio between the known alignment score and the highest substitution score [5]; the ratio is Ł/*α* with our scoring function. Accordingly, the maximum number of insertions and deletions is given by *m*−*L*/*α* for sequence *s*_*a*_ and *n*−*L*/*α* for sequence *s*_*b*_. This means that the matrix area to be filled out can be restricted within an anti-diagonal band: any cell (*i*,*j*) that satisfies −(*n*−*L*/*α*)≤*i*−*j*≤*m*−*L*/*α* must be filled out.

### GPU-based implementation

We implemented our method by extending SW# [5] such that it starts the forward matrix-filling phase using *L*=*L*_1_ and band optimization. SW# runs on at most two GPUs that are compatible with the compute unified device architecture (CUDA) [32]. This dual-GPU implementation divides the matrix *H* into two pieces, upper and lower, so that the pieces can be filled out in parallel. In other words, the lower piece is processed from the bottom right corner to the top left corner, while the upper piece is processed in the opposite direction.

Similar to CUDAlign 2.1 [16], SW# computes optimal local alignments in three phases. The first phase computes the highest alignment score and the ending alignment position. Our interpair optimization method is applied to the first phase, where neither the highest alignment score, the starting alignment position nor the ending alignment position is unknown before computation. The second phase processes a banded variant [24] of the Smith-Waterman algorithm with reverse subsequences that start from the computed ending position. This phase is also accelerated with a block pruning method that uses the highest alignment score instead of a lower bound. The final phase applies a modified version of the Myers-Miller algorithm [15] to the found subsequences. The modified version processes the Needleman-Wunsch algorithm [33] accelerated with Ukkonen’s band optimization [24].

SW# applies the pruning operation to matrix blocks rather than matrix cells. This block pruning optimization avoids thread divergence [32], which can significantly drop the execution efficiency on the single-instruction, multiple-thread (SIMT) architecture [32]. In other words, all cells (i.e., threads) in a block are computed or pruned, and thus, all threads in a warp have the same execution path after pruning.

Table 1 shows the specification of our experimental machine, which was equipped with two Tesla K40 GPUs. These GPUs are connected to PCIe 3.0 ×16 slots.

**Table 1:** Experimental machine

Item	Specification
CPU	Intel Xeon CPU E5-2680 v2 @ 2.80 GHz
Main memory	DDR3 512 GB
GPU	NVIDIA Tesla K40 ×2
Video memory	GDDR5 12 GB ×2
OS	Ubuntu 14.04.1
Compiler	gcc 4.8.2
Compiler option	O3
CUDA	CUDA 6.5

## Results and discussion

To evaluate our interpair optimization method in terms of execution time, we compared our method with the original SW#, which uses an existing intrapair pruning method [16]. Three variations were deployed for experiments: (1) an interpair pruned and banded version, (2) an interpair pruned version and (3) a banded version. We used the same scoring function as that employed by the other method [5, 16]: (*α*,*β*,*o*,*e*)=(1,−3,5,2).

Table 2 shows the specification of our experimental sequences [34]. Sequences *s*_1_– *s*_6_ are genomes of Bacillus anthracis. Sequences *s*_7_, *s*_8_ and *s*_9_ are genomes of the human chromosome 21. Sequences *s*_10_, *s*_11_ and *s*_12_ are genomes of the human chromosome 19, the gorilla chromosome 19 and the chimpanzee chromosome 19, respectively.

**Table 2:** Experimental datasets

Sequence	Accession number	Length (bp)	Remark
*s*_1_	[GenBank:CP002091]	5,218,947	Bacillus anthracis
*s*_2_	[GenBank:AE016879]	5,227,293	Bacillus anthracis
*s*_3_	[GenBank:CP001598]	5,227,419	Bacillus anthracis
*s*_4_	[GenBank:CP001970]	5,227,898	Bacillus anthracis
*s*_5_	[GenBank:AE017225]	5,228,663	Bacillus anthracis
*s*_6_	[GenBank:CP001215]	5,230,115	Bacillus anthracis
*s*_7_	[GenBank:AC_000153]	33,483,523	Human chromosome 21
*s*_8_	[GenBank:NC_000021]	46,709,983	Human chromosome 21
*s*_9_	[GenBank:NC_018932]	47,690,666	Human chromosome 21
*s*_10_	[GenBank:NC_000019]	58,617,616	Human chromosome 19
*s*_11_	[GenBank:FR853090]	56,181,278	Gorilla chromosome 19
*s*_12_	[GenBank:NC_006486]	63,644,993	Chimpanzee chromosome 19

### Comparison of execution time

**Fig. 7:**
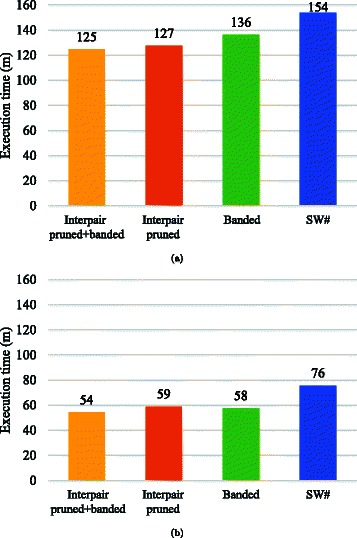
Execution times of the all-pairs comparisons. Results obtained from the implementations of (**a**) one GPU and (**b**) two GPUs

Figure 7 shows the execution time *T* spent for the all-pairs comparison of six base sequences: $\mathcal {S}=\{s_{1},s_{2},s_{3},s_{4},s_{5},s_{6}\}$. Execution time *T* includes the time spent for all three phases of SW#. Our method with interpair pruning and band optimization reduced the execution time from 154 to 125 m on a single GPU and from 76 to 54 m on dual GPUs. These results correspond to the speedups of 1.2 times and 1.4 times, respectively. Thus, our method was more effective on dual GPUs than on a single GPU.

**Fig. 8:**
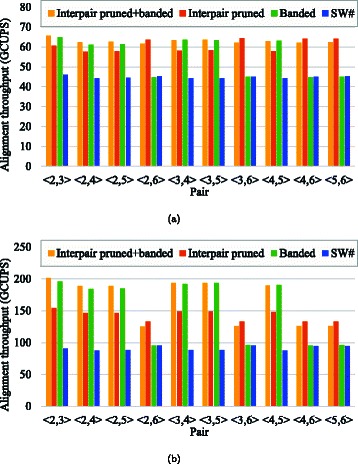
Alignment throughputs. Results obtained from the implementations of (**a**) one GPU and (**b**) two GPUs

To investigate the execution time in detail, we measured the alignment throughput *ρ*=(*m*+1)×(*n*+1)/*T* for all pairs. Figure 8 shows those measured throughputs in GCUPS. As can be seen, our method successfully increased the throughput for all pairs. The alignment throughputs on a single GPU were 62–66 GCUPS and those on dual GPUs were 126–202 GCUPS. Our dual GPU implementation achieved superlinear speedups that were 2.0–3.1 times faster than a single GPU implementation. Compared with the previous method’s speedups (which ranged from 2.0 to 2.1 times), our interpair optimization method can yield more efficient parallelization on dual-GPU systems.

### Pruning ratio

**Fig. 9:**
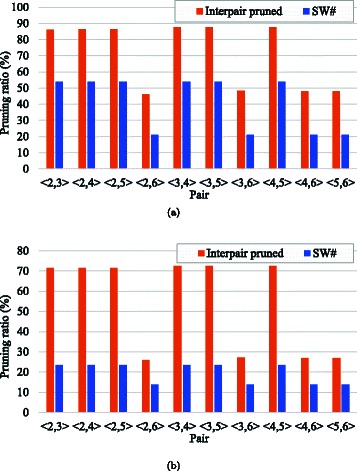
Pruning ratio. Results obtained from the implementations of (**a**) one GPU and (**b**) two GPUs

Figure 9 shows the pruning ratio *γ*=*c*/*m* *n*, where *c* represents the number of pruned matrix cells during forward matrix-filling (i.e., the first phase). Similar to the alignment throughput *ρ*, our method successfully increased the pruning ratio *γ* for all pairs. The maximum ratio on a single GPU was obtained for pair 〈3,4〉, which increased *γ* by 34 %. Similar results were obtained for other pairs. Thus, our interpair pruning method (which increased the pruning ratio) realized alignment throughput acceleration.

**Fig. 10:**
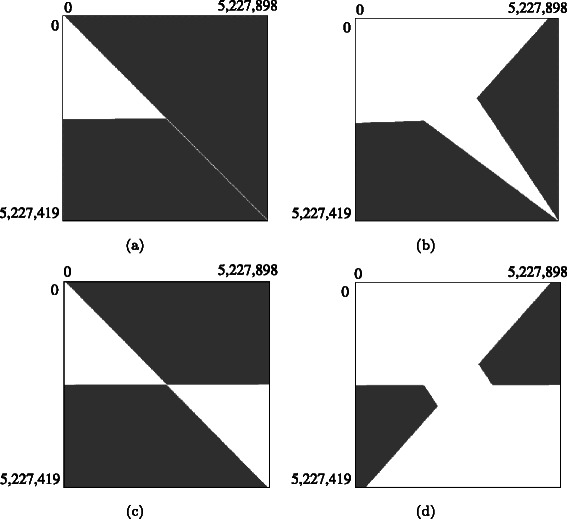
Pruned matrix cells for pair 〈3,4〉. (**a**) Proposed method on a single GPU, (**b**) previous method on a single GPU, (**c**) proposed method on dual GPUs, and (**d**) previous method on dual GPUs. Matrix cells in the gray area are pruned

The pruning ratio for pair 〈3,4〉 further increased from 34 % to 49 % on the dual-GPU implementation, which implies that because our method successfully increases the number of pruned matrix cells when using multiple GPUs, our pruning method is more effective on dual GPUs than on a single GPU. To analyze this behavior in depth, we investigated the distribution of pruned matrix cells. Figure 10 illustrates the area where matrix cells were pruned for pair 〈3,4〉; as that figure shows, our interpair method significantly enlarged the area of pruned matrix cells on dual GPUs.

As mentioned earlier, the previous intrapair method [16] can prune a matrix cell (*i*,*j*) that satisfies either *i*≥⌈*m*/2⌉ or *i*≥*n*−*j* on a single GPU. However, these conditions cannot be directly applied to dual GPUs because that parallel method divides the matrix *H* into two pieces on dual GPUs. Owing to this division, all matrix cells on the connecting border of these pieces must be computed to correctly integrate the local results into a global result; this restriction prohibits pruning a matrix cell that satisfies *i*≥⌈*m*/2⌉. Consequently, matrix cells that the previous method can prune satisfy either *i*≥*n*−*j* (in the upper piece) or *m*−*i*≥*j* (in the lower piece). Note that the latter condition can be easily derived by considering a piece turned upside down and right side left. In contrast, our method does not have this restriction because it uses a better lower bound that cannot be obtained by the intrapair pruning approach. Consequently, our interpair pruning approach is effective for achieving parallelization and efficient pruning with less computation.

**Fig. 11:**
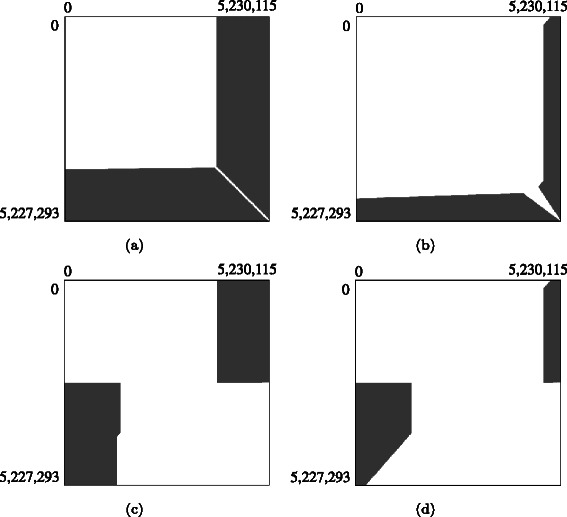
Pruned matrix cells for pair 〈2,6〉. (**a**) Proposed method on a single GPU, (**b**) previous method on a single GPU, (**c**) proposed method on dual GPUs, and (**d**) previous method on dual GPUs. Matrix cells in the gray area are pruned

We then analyzed how pruning was triggered during forward matrix-filling. There are four triggering patterns, each shaping a different border of the pruned area: (1) horizontal, (2) vertical, (3) diagonal, and (4) anti-diagonal. Horizontal and vertical borders appear when the highest score does not increase during the earlier phase of forward matrix-filling. Figure 11 shows an example of horizontal and vertical borders; in that example, the similar region of pair 〈2,6〉 existed in the latter part of the sequences. Consequently, the lower bound was rarely updated during the earlier phase of forward matrix-filling; thus, the triggering cells satisfy *i*=*m*−*d*_1_ or *j*=*n*−*d*_2_, where *d*_1_ and *d*_2_ are constant values. Diagonal borders appear around the highest scoring cell because its right and bottom neighbors can be pruned; such borders can be observed in our method and the previous method. Finally, anti-diagonal borders appear when the lower bound increases during forward matrix-filling. Anti-diagonal borders appear in the area that satisfies *i*≥*n*−*j* and *i*≤⌈*m*/2⌉, as shown in Fig. 10(b). Our method failed to update the lower bound within this region; consequently, anti-diagonal borders were observed only in the previous method.

Next, we investigated the alignment results of the best and worst cases in terms of speedup. As shown in Table 3, the best and worst speedups on a single GPU were obtained for pairs 〈3,6〉 and 〈2,5〉, respectively. The lower bound of the best case was *L*=1,433,837, which was the fourth smallest value among possible pairs; with that lower bound, our method increased the pruning ratio from 21 % to 48 % (a 2.3 times higher ratio), which was the best pruning ratio improvement obtained. In contrast, the lower bound of the worst case was *L*=5,061,056, which was the fourth largest value among possible pairs; with that lower bound, the pruning ratio increased from 53 % to 86 % (1.6 times), which was the third smallest ratio improvement. Thus, our method’s impact depends on the pruning ratio of the intrapair pruning method. Our method is effective if the original pruning ratio (obtained with intrapair pruning) is relatively small; however, its effectiveness is limited if the original pruning ratio is high.

**Table 3:** Summary of the alignment results. With respect to the genomes of Bacillus anthracis, the best and worst speedups on a single GPU were obtained for pairs 〈3,6〉 and 〈2,5〉, respectively. Because our method estimates a lower bound from aligned pairs, early-processed pairs such as 〈1,2〉, 〈1,5〉, 〈7,8〉, 〈7,9〉, 〈10,11〉, and 〈10,12〉 use *L*=0. In contrast, our method fails to increase the initial lower bound for pair 〈11,12〉

Pair	Initial lower bound *L*	Score	# of mismatches	# of gaps	Similar region	
					[*x*, *y*]	[*z*, *w*]
〈2,3〉	5,054,849	5,226,806	19	134	[101, 5,227,293]	[1, 5,227,319]
〈2,6〉	1,360,803	1,439,963	234	1,991	[3,781,820, 5,227,293]	[3,783,219, 5,229,989]
〈3,6〉	1,433,837	1,440,080	231	1,990	[3,781,847, 5,227,419]	[3,783,219, 5,230,089]
〈1,2〉	0	5,179,709	628	15,972	[1, 5,218,947]	[1, 5,227,293]
〈1,5〉	0	5,183,765	657	14,578	[1, 5,218,946]	[2, 5,228,663]
〈2,5〉	5,061,056	5,220,960	165	2,430	[1, 5,227,292]	[2, 5,228,663]
〈7,8〉	0	31,073,252	178,471	425,571	[570,587, 33,483,523]	[13,789,327, 46,683,588]
〈7,9〉	0	30,779,997	168,946	472,978	[799,132, 33,483,523]	[14,990,367, 47,664,260]
〈8,9〉	26,801,979	32,682,564	113,078	238,057	[12,915,809, 46,709,983]	[13,921,377, 47,690,666]
〈10,11〉	0	6,268,702	3,664,543	3,655,628	[27,961,827, 58,340,489]	[25,036,812, 55,918,720]
〈10,12〉	0	14,383,541	1,626,256	3,780,342	[27,437,780, 58,535,035]	[32,521,523, 63,596,428]
〈11,12〉	0	3,113,889	636,188	1,032,735	[25,014,281, 33,464,562]	[33,028,220, 41,362,068]

We also conducted experiments using the three genomes of the human chromosome 21: sequences *s*_7_, *s*_8_ and *s*_9_. For pair 〈7,8〉, the similar region on sequence *s*_7_ was [570,587, 33,483,523]. However, the similar region was [799,132, 33,483,523] for pair 〈7,9〉. According to these alignment results, we obtained a lower bound *L*=26,801,979 on the score for pair 〈8,9〉. This lower bound successfully increased the pruning ratio for pair 〈8,9〉 from 40 % to 73 % on a single GPU and from 16 % to 53 % on a dual GPU. Accordingly, we achieved the speedups of 1.2 and 1.3 times on a single GPU and dual GPUs, respectively.

### Case study with different species

We next performed experiments to evaluate the effectiveness of our method for different species. Genomes of the human chromosome 19, chimpanzee chromosome 19 and gorilla chromosome 19 were aligned: sequences *s*_10_, *s*_11_, and *s*_12_ in Table 2.

Table 3 summarizes the alignment results. For pair 〈10,11〉, the similar region on the human sequence 10 was [ 27,961,827,58,340,489]. For pair 〈10,12〉, that similar region was [ 27,437,780,58,535,035]. Thus, there was a common part between the two pairs. However, the lower bound computed from these results was *L*=−35,205,808<0. Because this lower bound is smaller than zero (which was the initial value used in the previous method), our interpair optimization method failed to increase the number of pruned matrix cells. As shown in Table 3, many gaps were needed to align the sequences of these different species; thus, these dissimilar sequences resulted in *L*<0. Because the opening penalty *o* is set to five, *L* becomes a negative value if gaps occupy more than 20 % of the common part.

The abovementioned negative value indicates that our assumption on the worst case, where all mismatches and gaps occur in the common part of similar regions, is too strict to improve the lower bound for different species. Consequently, we think that the lower bound can be improved by relaxing this worst case. For example, statistical information such as the distribution of symbols may be useful to relax this assumption.

### Applicability

**Fig. 12:**
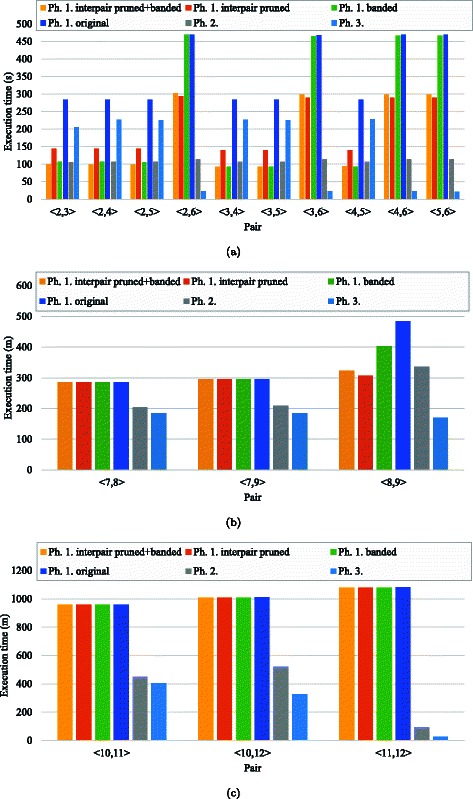
Breakdown analysis. Breakdown of execution time for genomes of (**a**) Bacillus anthracis, (**b**) the human chromosome 21 and (**c**) the human/gorilla/chimpanzee chromosome 19. The original version of phase 1 corresponds to the score-only Smith-Waterman algorithm with intrapair pruning. Phases 2 and 3 correspond to a banded (score-only) Smith-Waterman algorithm with block pruning and a banded Myers-Miller algorithm, respectively

Finally, we evaluated our interpair optimization method in terms of the applicability. To do this, we compared our method with banded alignment algorithms employed in the second and third phases of SW#. Figure 12 shows the breakdown of execution time for experimental datasets. Notice that the three phases usually examine different lengths of subsequences, because the first and second phases find the ending and starting alignment positions, respectively; the subsequences to be examined become shorter as the alignment phase proceeds.

As shown in Fig. 12(a), phase 2 took around 110 s to execute the banded Smith-Waterman algorithm for genomes of Bacillus anthracis. In contrast, execution times of phase 1 ranged from 93 s to 470 s, depending on the pruning ratio. The shortest time of 93 s was achieved by our interpair pruned and banded version that achieved the best pruning ratio of 88 %. As compared with the banded Smith-Waterman algorithm (phase 2), our pruned version (without band optimization) took 1.3–2.6 times longer execution time. These results indicate that execution time is mainly dominated by the length of the optimal local alignment rather than the width of the specified band. In Fig. 12(a), irregular behaviors can be seen at pairs 〈2,6〉, 〈3,6〉, 〈4,6〉 and 〈5,6〉; the banded version took almost the same execution time as the original version. These pairs have relatively short local alignments as compared with the remaining pairs (see Table 3). Because the first phase tried to fill out the entire matrix, the band was not narrow enough to achieve acceleration over the original version. Notice that this situation was avoided at the last phase, where the found starting and ending alignment positions reduced the matrix size; the rectangle area to be filled out was tightly bounded before computation.

The execution times of the last phase can be classified into two groups: a group around 20 s and that around 230 s. This large gap between 20 s and 230 s was due to the length of the optimal local alignment. The former group obtained four times longer local alignments than the latter. Because the time complexity of the Myers-Miller algorithm is $\mathcal {O}(mn)$, the former group can compute 16 times more matrix cells than the latter group.

Similar behaviors were observed with long sequences except pair 〈8,9〉. As shown in Fig. 12(b), phase 2 took longer time than phase 1 (interpair pruned). This timing result implies that our pruning method is efficient against the increase of the sequence length. As compared with pairs 〈7,8〉 and 〈7,9〉, pair 〈8,9〉 deals with a 1.4 times longer sequence (see Table 2). This longer sequence increased the execution time of phase 2 and that of phase 1 (original) by ×1.65 and ×1.69, respectively. In contrast, that of phase 1 (interpair pruned) increased by ×1.07, demonstrating an efficient pruning effect. In fact, our interpair method pruned 73 % of matrix cells for pair 〈8,9〉, whereas the original intrapair method pruned 50 % of matrix cells for pairs 〈7,8〉 and 〈7,9〉.

In summary, banded alignment algorithms achieved shorter execution time than pruning-based alignment algorithms. However, band optimization can be inefficient if the length of the local alignment is relatively short as compared with that of sequences. This inefficiency can fail to accelerate the first phase, where the length of the local alignment is unknown before computation. On the other hand, pruning-based alignment algorithms, which avoid unnecessary computation at runtime, are useful to deal with this performance issue.

## Conclusions

An interpair optimization method has been presented for accelerating the all-pairs SW comparisons of sequences. Based on the alignment results of the compared pairs, our method computes a lower bound on the similarity score for other pairs that have not yet been aligned. This lower bound is then used as the initial lower bound to increase the efficiency of an existing intrapair pruning method that is capable of reducing the execution time of the matrix-filling phase. The lower bound is further used for band optimization. We have also proven that the computed lower bound is larger than the optimal solution.

Experimental results show that our interpair optimization method when running on a single GPU is 1.2 times faster than an intrapair pruning method. This speedup further increased to 1.4 times when run on dual GPUs. The maximum pruning ratio was 88 % on a single GPU and 49 % on dual GPUs. However, for the sequences of different species, our method failed to improve the initial lower bound; thus, that acceleration over the intrapair pruning method was not achieved. This failure was because of the many gaps that needed to be aligned between such dissimilar sequences.

Future study includes an application to multi-node systems such as CUDAlign 3.0 [17], which runs on a 64-node cluster of GPUs.

## References

[1] Smith TF, Waterman MS (1981). Identification of common molecular subsequences. J Mol Biol.

[2] Manavski SA, Valle G (2008). CUDA compatible GPU cards as efficient hardware accelerators for Smith-Waterman sequence alignment. BMC Bioinforma.

[3] de O. Sandes EF, de Melo ACMA. CUDAlign: Using GPU to accelerate the comparison of megabase genomic sequences. In: Proc. 15th ACM SIGPLAN Symp. Principles and Practice of Parallel Programming (PPoPP’10). ACM: 2010. p. 137–46.

[4] Blazewicz J, Frohmberg W, Kierzynka M, Pesch E, Wojciechowski P (2011). Protein alignment algorithms with an efficient backtracking routine on multiple GPUs. BMC Bioinforma.

[5] Korpar M, Šikić M (2013). Sw#–gpu-enabled exact alignments on genome scale. Bioinformatics.

[6] Farrar M (2007). Striped Smith-Waterman speeds database searches six times over other SIMD implementations. Bioinformatics.

[7] Szalkowski A, Ledergerber C, Krähenbühl P, Dessimoz C (2008). SWPS3 — fast multi-threaded vectorized Smith-Waterman for IBM Cell/B.E. and x86/SSE2. BMC Res Notes.

[8] Rognes T (2010). Faster Smith-Waterman database searches with inter-sequence SIMD parallelisation. BMC Bioinforma.

[9] Li IT, Shum W, Truong K (2007). 160-fold acceleration of the Smith-Waterman algorithm using a field programmable gate array (FPGA). BMC Bioinforma.

[10] Liu Y, Schmidt B. SWAPHI: Smith-Waterman protein database search on Xeon Phi coprocessors. In: Proc. 25th IEEE Int’l Conf. Application-specific Systems, Architectures and Processors (ASAP’14). IEEE: 2014. p. 184–5.

[11] Lindholm E, Nickolls J, Oberman S, Montrym J (2008). NVIDIA Tesla: A unified graphics and computing architecture. IEEE Micro.

[12] Owens JD, Houston M, Luebke D, Green S, Stone JE, Phillips JC (2008). GPU computing. Proc IEEE.

[13] Ino F, Munekawa Y, Hagihara K (2012). Sequence homology search using fine grained cycle sharing of idle GPUs. IEEE Trans Parallel Distrib Syst.

[14] Ikeda K, Ino F, Hagihara K (2014). Efficient acceleration of mutual information computation for nonrigid registration using CUDA. IEEE J Biomed Health Informa.

[15] Myers EW, Miller W (1988). Optimal alignments in linear space. Comput Appl Biosci.

[16] de O. Sandes EF, de Melo ACMA (2013). Retrieving Smith-Waterman alignments with optimizations for megabase biological sequences using GPU. IEEE Trans Parallel Distrib Syst.

[17] de O. Sandes EF, Miranda G, de Melo ACMA, Martorell X, Ayguadé E. Cudalign 3.0: Parallel biological sequence comparison in large gpu clusters. In: Proc. 14th IEEE/ACM Int’l Symp. Cluster, Cloud and Grid Computing (CCGrid’14). IEEE: 2014. p. 160–9.

[18] Feng DF, Doolittle RF (1987). Progressive sequence alignment as a prerequisite to correct phylogenetic trees. J Mol Evol.

[19] Chao KM, Pearson WR, Miller W (1992). Aligning two sequences within a specified diagonal band. Comput Appl Biosci.

[20] Thompson JD, Higgins DG, Gibson TJ (1994). CLUSTAL W: improving the sensitivity of progressive multiple sequence alignment through sequence weighting, position-specific gap penalties and weight matrix choice. Nucleic Acids Res.

[21] Notredame C, Higgins DG, Heringa J (2000). T-Coffee: A novel method for fast and accurate multiple sequence alignment. J Mol Biol.

[22] Bashford D, Chothia C, Lesk AM (1987). Determinants of a protein fold: Unique features of the globin amino acid sequences. J Mol Biol.

[23] Blazewicz J, Frohmberg W, Kierzynka M, Wojciechowski P (2013). G-MSA–A GPU-based, fast and accurate algorithm for multiple sequence alignment. J Parallel Distrib Comput.

[24] Ukkonen E (1985). Algorithms for approximate string matching. Inf Control.

[25] Klimovitski A. Using SSE and SSE2: Misconceptions and reality. In: Intel Developer Update Magazine. Intel: 2001.

[26] Intel Corporation: Intel Xeon Phi Product Family. http://www.intel.com/content/www/us/en/processors/xeon/xeon-phi-detail.html. Accessed 18 Sep 2015.

[27] Liu W, Schmidt B, Voss G, Müller-Wittig W (2007). Streaming algorithms for biological sequence alignment on GPUs. IEEE Trans Parallel Distrib Syst.

[28] Liu Y, Schmidt B, Maskell DL (2010). CUDASW++2.0: enhanced Smith-Waterman protein database search on cuda-enabled GPUs based on SIMT and virtualized SIMD abstractions. BMC Res Notes.

[29] Munekawa Y, Ino F, Hagihara K (2010). Accelerating Smith-Waterman algorithm for biological database search on CUDA-compatible GPUs. IEICE Trans Inf Syst.

[30] Gotoh O (1982). An improved algorithm for matching biological sequences. J Mol Biol.

[31] Hirschberg DS (1975). A linear space algorithm for computing maximal common subsequences. Commun ACM.

[32] NVIDIA Corporation. CUDA C Programming Guide Version 6.5. 2014. http://docs.nvidia.com/cuda/pdf/CUDA_C_Programming_Guide.pdf. Accessed 18 Sep 2015.

[33] Needleman SB, Wunsch CD (1970). A general method applicable to the search for similarities in the amino acid sequence of two proteins. J Mol Biol.

[34] National Center for Biotechnology Information: NCBI data. 2014. http://www.ncbi.nlm.nih.gov/. Accessed 18 Sep 2015.

